# Nursing management in Santander: Own knowledge or health administration?

**DOI:** 10.15649/cuidarte.3067

**Published:** 2023-09-06

**Authors:** Diana Isabel Cáceres-Rivera, Mayerli Katherine Rincón-Romero

**Affiliations:** 1 Universidad Cooperativa de Colombia, Bucaramanga, Colombia. Email: dianai.caceres@ucc.edu.co Universidad Cooperativa de Colombia Universidad Cooperativa de Colombia Bucaramanga Colombia dianai.caceres@ucc.edu.co; 2 Universidad Cooperativa de Colombia, Bucaramanga, Colombia. E-mail: mayerli.rincon@campusucc.edu.co Universidad Cooperativa de Colombia Universidad Cooperativa de Colombia Bucaramanga Colombia

Health needs remain an issue of interest for the comprehensive development of countries. Defining actions to respond to the new environmental, political, and social scenarios and challenges of today's world is a priority. Nursing care management establishes a leading process within the interventions based on the scenarios proposed by the World Health Organization (WHO) and the Pan American Health Organization (PAHO)^1^ for this development.

There are many definitions of care management. One defines it as the ability to plan, organize, motivate, and control the provision of timely, safe, comprehensive care that ensures continuity of care and is based on clear guidelines to achieve the ultimate goal of improving the health of individuals^2^; it also aims to adapt care to the needs of the individual, the requirements of family members, caregivers, and the community^3^^,^^4^. This includes working together, where consulting with nursing colleagues creates networks with active participation in advancing knowledge and interdisciplinary collaboration, achieving an impact on the quality of service they provide, as well as strengthening the potential and enriching meaningful learning in professional practice^5^.

Over time, the nursing discipline has evolved its knowledge and moved toward a systematic, step-by-step approach, creating its own framework for action called the “Nursing Care Process” (NCP)-a tool that has proven effective in managing resources to support care management. In addition, it uses evidence-based nursing (EBN) as a resource for making the best decisions in health promotion, diagnosis, treatment, rehabilitation, or palliative care.

## Health administration and management of care

In light of organizational management, the fundamental objective is to establish management models in companies that allow them to offer products or services that meet users' needs in a competitive environment. In this sense, it is not enough to perform tasks correctly; it is necessary to outperform the competitors by implementing new innovative strategies of the organization's management^6^.

Care management is a major challenge in terms of managerial and administrative knowledge governed by regulations. To achieve quality care, it is necessary to have scientific competence in the care and processes to be managed. Managing care requires competence in administration, critical judgment, vision, foresight, and the ability to respond to rapid changes in health and disease, as well as the effective use of information for cooperation and coordination of work. In nursing, this appropriate managerial competency is developed based on the ability to manage resources, plan care, coordinate, supervise, and control^7^^,^^8^^,^^9^.

We must consider strategies to improve nursing care and ensure the continuity of patient-centered care by developing organizational models and clinical practices. To achieve this, it is important to implement different tools to ensure quality and a useful methodology for the nursing process as a scientific method for elaborating and developing care plans.

More than a hundred years ago, nursing began to develop its theoretical underpinnings through research studies. Florence Nightingale reflected on her observations and collected and analyzed data in the hospitals where she worked to improve health services. However, it was not until the second half of the last century that nursing demonstrated its systematic study through rigorous research and the formulation of several theoretical models and theories to support practice^10^.

Understanding nursing conceptual models can be useful in nursing practice by allowing us to organize our thoughts about different aspects of practice situations. In addition, the use of common terminology among professionals can facilitate meaningful communication. It can also serve as a guide for care, teaching, and research practice^11^.

Therefore, for nursing care management to be complete and more comprehensive, it is necessary to understand and appreciate the frameworks used in care management, and how they guide professional and disciplinary development. Paying attention to environmental challenges helps the professions maintain up-to-date and quality care, but above all, based on solid foundations.

In this sense, from the perspective of Orem's self-care theory, the relevant elements of nursing care are highlighted. The proposal seeks to direct and manage nursing care to assist individuals in achieving and maintaining self-care actions that promote health, life, recovery from illness, and adaptation to the effects of illness^12^^,^^13^^,^^14^. Within this disciplinary framework, four nursing theorists-Imogene King, Virginia Henderson, Dorothea Orem, and Jean Watson-include elements that guide the management function in their postulates. In Imogene King's approach, hospitals are the most important environmental factor for the person receiving institutional care. For Henderson, recognizing individual needs is paramount. In the case of Orem, in addition to the above, it is essential to ensure the availability of adequate resources for care. Watson argues that the nursing approach is intrinsically linked to human caring in all areas of professional practice, including administration, management, teaching, service, and research^15^.

However, recognizing nurses' skills contributes to constructing a different scientific paradigm that challenges fear and takes risks in the face of the challenges of caring.

## Tools for management of care in Santander

Evidence-based nursing contributes to the demand for new management approaches focusing on personalized care based on individual health experiences. The following are the advances in care management in the Department of Santander found in the literature.

One of the first publications on applying nursing tools, such as the nursing process, was published in 1997. It was a descriptive study that collected data on nurses' knowledge and applicability of the nursing process in Bucaramanga and its metropolitan area. The study found that only 25% of the nurses correctly recalled the stages of the nursing process. Important limitations in the implementation of the nursing process were described, such as the time required for its application and recording, the lack of consensus in the elaboration of the diagnosis, the lack of institutional commitment to adopt the process as a working tool, the general lack of knowledge of the process among the staff, the absence of specific record systems, and the patients' burden on nurses in relation to the number of nurses available. The article's authors proposed solutions to overcome these limitations, including continuing education programs, unification of criteria among professionals and teachers, increasing the number of nurses in institutions, institutional support to create specific registration systems, and more economic resources for health care institutions^16^.

Twenty-four years later, in 2018, Lesmes studied the factors associated with the application of the nursing process, and 59% of participants reported using it. However, 98% reported lack of time as a factor for not applying it. A statistically significant relationship was found between the use of the nursing process and its perception as a facilitating tool for care, and the nurse's ability to relate it to the theory learned at the university. Unlike previous studies, these associations were not limited to working or academic conditions. The results suggest the importance of applying theory to practice and evaluating its implementation in different professional settings^17^.

In 2008, the Hospital Universitario de Santander published one of the first reports describing care management improvement processes. The study presented the results of implementing a quality control model of nursing care aimed at identifying the strengths and weaknesses of the nursing care process, proposing corrective, preventive, and improvement actions, and developing new evidence-based approaches to care to make appropriate changes. This process consisted of designing nursing processes and protocols that included nursing records, hand hygiene, peripheral venous catheterization, adverse events, and change-of-shift handoffs. With this implementation, they concluded that they had improved the quality of service, patient and family satisfaction, efficient use of resources, and optimization of the work of nurses and nursing assistants to strengthen the teaching-assistance processes^18^.

The Fundación Oftalmológica de Santander Clínica Carlos Ardila Lulle (FOSCAL) was identified as another institution that has implemented care management improvement processes by adopting some of the Registered Nurses' Association of Ontario (RNAO) Good Practice Guidelines (BPG Program), which the Ontario Ministry of Health has funded for several years. Since their inception, these guidelines' purpose has been to assist nurses by providing evidence-based guidelines for patient care in various healthcare settings and sectors^19^. In the case of FOSCAL, it has implemented specific guidelines for pain assessment and management, prevention of falls and fall injuries, and the risk of skin pressure injuries. In a recent article on the sustainability of this model, the authors report 89% adherence to the guidelines. There are no published data yet on the effectiveness of this model concerning quality indicators^20^.

Clearly, providing adequate nursing care management at all life cycle stages is a significant challenge, where nursing research in the different areas of professional performance is fundamental to strengthening evidence based practice and effectiveness in care processes. In this regard, another institution that has contributed to care management is the Fundación Cardiovascular de Colombia (FCV), which has published instrument validations to contribute to the culture of quality and patient safety^21^^,^^22^.

## Care management needs identified in the region.

One of the most felt needs in care management is health education for patients and their families. Continuing to innovate in this type of intervention is still at the forefront since, despite the efforts constantly being made in this subject, a lack of knowledge has been identified in up to 80% of the users of the same institution^23^. Similarly, this situation has also been studied among nurses, where, for example, in the area of law, in addition to the need for training processes, deficient levels of knowledge have been found^24^. In the nursing profession, care is based on a communicative act that involves the application of scientific and technological knowledge as well as understanding the socio-cultural context in which patient care is provided. It is there that the substantive processes of constant updating emerge, and it is a priority to advance to high-level post-graduate education: master's degrees and doctorates.

In nursing practice, the processes of organizing, supervising, evaluating, and promoting the quality of care need to be unveiled, and the perception of nursing performance is an issue that has become relevant in recent years. In this regard, some studies have attempted to identify strengths and weaknesses, such as the study by Valdivieso, which, although old, is one of the first descriptions of nurses in Santander. In the study, most nurses in the country, especially those in the eastern, southeastern, and coastal regions, showed a positive attitude toward their professional practice. However, the unfavorable perception has to do with a lack of social recognition and unfavorable attitudes towards autonomy and trade union organization in the country^25^. This situation highlights the urgent need for actions that promote leadership and change at the union and institutional levels, implement care strategies with a disciplinary basis, and promote processes to improve healthcare quality.

This issue has been mentioned for several years. In 2007, Beatriz Carvallo, then president of the National Association of Nurses of Colombia (ANEC, for its acronym in Spanish), spoke at the National Congress of Nurses about the impact of social security reforms on nurses' social and working conditions. Since then, there have been situations that have become challenges, such as the reduction of nursing autonomy and the disappearance of problems in nursing departments, the substitution of nursing functions by administrative ones, and the disregard of the WHO recommendation to strengthen nursing. This is the real challenge for nursing care management. Regarding this scenario, regional studies have been described concerning the workload of nurses, where the need to evaluate and analyze the distribution of human resources in these units is evident^26^. These issues are relevant because Santander is a reference in the health sector in northeastern Colombia and South America. Medical tourism is an important part of the region's economy, so nursing should contribute to social development through its disciplinary work.

From a qualitative perspective, in some settings, such as inpatient services, there were negative comments about the management of human caring. Nursing care can be improved by spending more time listening to patients, providing information about patients' care and condition, providing timely care, improving the nurse's mood, and providing appropriate care during night hours^27^. These results justify the need to publish everything planned and evaluated by management to validate the leadership exercised by the nursing departments and directorates. However, it is clear that many of these difficulties are related to the lack of implementation of nursing care models.

Regarding this leadership, a study conducted in 2010 in a group of 107 nurses from two health institutions in Bucaramanga, using the Hersey and Blanchard instrument, described a predominant leadership style of guiding and participating, which means that there is a high emphasis in relationship and low emphasis in tasks, respectively. The author emphasizes the importance of leadership in nursing education to integrate skills such as decision making, leadership, communication, continuing education, and administration. With their skills and training, they will continue to be of great value to healthcare organizations^28^.

Finally, the goal of nursing is to care, and the goal of management is to direct the production of goods or services in organizations^8^. This suggests that care management is becoming important, and that leadership and evidence-based decision-making skills are challenging.
