# Supplementary material for: Nursing management in Santander: Own knowledge or health administration?
Source: Rev Cuid. 2023 Sep 6;14(2):e01. doi: 10.15649/cuidarte.3067 (PMC11559330; doi:10.15649/cuidarte.3067)
Supplement: Translation

# Translation: Gestión en Enfermería en Santander: ¿conocimiento propio o administración en salud?

Las necesidades en salud permanecen como un tema de interés en el desarrollo integral de los países. Es prioritario establecer acciones para dar respuesta a los nuevos escenarios y retos ambientales, políticos y sociales del mundo actual. La gestión del cuidado de enfermería establece un proceso protagónico dentro de las intervenciones basadas en los escenarios propuestos de la Organización Mundial de la Salud (OMS) y la Organización Panamericana de la Salud (OPS)^1^ para este desarrollo.

Existen varias definiciones sobre gestión del cuidado, en una de estas se precisa como la capacidad de planear, organizar, motivar y controlar la provisión de cuidados oportunos, de manera segura, integral, que garanticen la continuación de la atención y están basados en lineamientos claros para obtener como fin último la mejoría de la salud de las personas^2^; tiene además el propósito de adecuar el cuidado a las necesidades de la persona, las demandas de sus familiares, cuidadores y la comunidad^3^^,^^4^. Esto incluye el trabajo en conjunto, donde la consulta entre colegas profesionales de enfermería genere redes con participación activa en el avance de conocimientos y la colaboración interdisciplinaria logrando un impacto en la calidad del servicio que están prestando, además de reforzar el potencial y enriquecer el aprendizaje significativo en la práctica profesional^5^.

En el transcurso del tiempo, la disciplina de enfermería ha evolucionado en su conocimiento, avanzando hacia un paso a paso sistemático, generando un marco de acción propio denominado “Proceso de Atención de Enfermería” (PAE), herramienta que ha demostrado contribuir de forma eficaz en la administración de recursos para favorecer la gestión del cuidado. Además, hace uso de la Enfermería basada en le evidencia (EBE), como recurso para tomar las mejores decisiones en acciones desde la promoción de salud, diagnóstico, tratamiento, rehabilitación o cuidados paliativos.

## Administración en Salud y Gestión del Cuidado

A la luz de la gestión organizacional, el propósito fundamental es establecer en las empresas modelos de gestión que permitan ofrecer productos o servicios que satisfagan las necesidades de los usuarios en un entorno competitivo. En este sentido, el simple hecho de realizar las tareas correctamente no es suficiente, es necesario superar a la competencia mediante la implementación de nuevas estrategias innovadoras desde la dirección de la organización^6^.

La gestión del cuidado representa un gran desafío en términos de conocimientos gerenciales y administrativos que están regidos por normativas. Para lograr una atención de calidad es necesario contar con competencias científicas en cuanto a los cuidados y los procesos que se deben gestionar. La gestión del cuidado implica una competencia en la administración y requiere juicio crítico, visión, previsión y capacidad de reacción ante los rápidos cambios en la salud y la enfermedad, así como el uso efectivo de la información para la cooperación y coordinación del trabajo. En enfermería, el desarrollo de esta capacidad gerencial adecuada se basa en habilidades para administrar recursos, planificar cuidados, coordinar, supervisar y controlar^7^^,^^8^^,^^9^.

Debemos considerar estrategias para mejorar la atención de enfermería y garantizar la continuidad del cuidado centrado en el paciente a través del desarrollo de modelos organizativos y prácticas clínicas. Para lograr esto, es importante implementar diferentes herramientas que aseguren la calidad, y una metodología útil es el proceso enfermero como método científico para la elaboración y desarrollo de planes de cuidado.

Hace más de cien años, la enfermería comenzó a desarrollar su sustento teórico a través de estudios de investigación. Florence Nightingale reflexionó sobre sus observaciones, recolectó y analizó datos en los hospitales donde trabajaron para mejorar los servicios de salud. Aunque no fue hasta en la segunda mitad del siglo pasado cuando la enfermería demostró concretar su estudio sistemático mediante la investigación rigurosa y la formulación de varios modelos teóricos y teorías que sustentan la práctica^10^.

Por consiguiente, la comprensión de los modelos conceptuales de enfermería puede ser útil en la práctica asistencial al permitirnos organizar nuestros pensamientos sobre los diferentes aspectos de una situación práctica. Además, el uso de una terminología común entre los profesionales puede facilitar la comunicación significativa. También puede servir como una guía en la práctica asistencial, docente e investigadora^11^.

En concordancia, para que la gestión del cuidado de enfermería sea más completa y global, es necesario comprender y valorar: los marcos de referencia utilizados en la gestión del cuidado y cómo orientan el desarrollo profesional y disciplinario. Prestar atención a los desafíos presentes en el entorno ayuda a las profesiones a mantener una atención actualizada y de calidad, pero sobre todo basado en fundamentos sólidos.

En este sentido, desde la teoría de autocuidado de Orem, se destacan los elementos relevantes para la atención de enfermería. La propuesta busca dirigir y administrar los cuidados de enfermería, con la finalidad de asistir al individuo en el logro y mantenimiento de sus acciones de autocuidado, favoreciendo así la salud, la vida, la recuperación de la enfermedad y la adaptación a sus efectos^12^^,^^13^^,^^14^. En este marco disciplinar, cuatro teóricas de enfermería: Imogene King, Virginia Henderson, Dorothea Orem y Jean Watson, incluyen en sus postulados elementos que orientan la función gerencial. En el enfoque de Imogene King, la institución hospitalaria representa el factor ambiental más relevante para la persona que recibe cuidados institucionales. Para Henderson, es primordial reconocer las necesidades individuales. En el caso de Orem, además de lo anterior, es esencial garantizar la disponibilidad de recursos adecuados para el cuidado. Watson sostiene que el abordaje de la enfermería se encuentra intrínsecamente ligado al cuidado humanizado en todas las áreas de la práctica profesional, incluyendo la administración, gestión, docencia, servicio e investigación^15^.

Ahora bien, el reconocimiento de las habilidades del profesional de enfermería contribuye a la construcción de un distinto paradigma científico, retando el miedo y asumiendo los riesgos frente a los desafíos de cuidar.

## Herramientas en la Gestión del Cuidado en Santander

Desde la enfermería basada en la evidencia, se contribuye a la demanda de nuevos enfoques de gestión orientados hacia la atención personalizada y basada en la experiencia de salud individual. A continuación, se exponen los avances encontrados en la literatura acerca de la gestión de cuidado en el departamento Santander.

Uno de las primeras publicaciones que se realizó sobre aplicación de herramientas propias de enfermería como lo es el proceso de enfermería fue hecha en 1997 en donde en un estudio descriptivo se establecieron datos acerca su conocimiento y aplicabilidad por parte de profesionales de enfermería en Bucaramanga y su área metropolitana, encontrándose que solo un 25% de los enfermeros recordaba correctamente las etapas. Se describieron limitaciones importantes para la implementación del proceso de enfermería, tales como el tiempo requerido para su aplicación y registro, la falta de consenso en la elaboración del diagnóstico, la falta de compromiso institucional para adoptar el proceso como una herramienta de trabajo, el desconocimiento generalizado del mismo por parte del personal, la ausencia de sistemas de registro específicos y la sobrecarga de pacientes en relación con el número de enfermeras disponibles. Los autores del artículo propusieron soluciones para superar estas limitaciones, incluyendo programas de educación continua, la unificación de criterios entre profesionales y docentes, un aumento en la cantidad de enfermeras en las instituciones, lograr el apoyo institucional para crear sistemas de registro especifico y destinar mayores recursos económicos para las instituciones de salud^16^.

Consecutivamente, 24 años después, Lesmes en el año 2018 estudió los factores relacionados con la aplicación del proceso de enfermería en donde el 59% de los participantes expresaron su uso. Sin embargo, un 98% refirió la falta de tiempo como factor para no aplicarlo. Se encontró una relación estadísticamente significativa entre la aplicación del proceso de atención de enfermería y su percepción como una herramienta facilitadora del cuidado, así como con la capacidad del profesional de enfermería de relacionarla con la teoría aprendida en la universidad. A diferencia de estudios previos, estas asociaciones no se limitaron las condiciones laborales o académicas. Los resultados sugieren la importancia de aplicar la teoría en la práctica y evaluar su implementación en diversos entornos profesionales^17^.

En 2008, el Hospital Universitario de Santander publicó uno de los primeros informes que describía procesos de mejora en la gestión del cuidado. El estudio presentó los resultados de la implementación de un modelo para el control de calidad del cuidado de enfermería, el cual tenía como objetivo detectar las fortalezas y debilidades del proceso de atención de enfermería, proponer medidas correctivas, preventivas y de mejora, así como desarrollar nuevos enfoques de cuidado basados en la evidencia para realizar cambios adecuados. Este proceso consistió en el diseño de procesos y protocolos para enfermería que incluyeron temas como registros de enfermería, lavado de manos, cateterismo venoso periférico, eventos adversos y entrega y recibo de turno. Con esta implementación concluyeron lograr una mejora en la calidad del servicio, la satisfacción del enfermo y los familiares, haber hecho uso eficiente de los recursos, optimizar el trabajo de las enfermeras y auxiliares del hospital a fin de fortalecer los procesos docencia-asistencia^18^.

Se encontró que, La Fundación Oftalmológica de Santander Clínica Carlos Ardila Lulle (FOSCAL) es otra institución que ha implementado procesos de mejora en la gestión del cuidado mediante la adopción de algunas de las Guías de Buenas Prácticas (BPG Program) de la RNAO (Registered Nurses' Association of Ontario) financiadas por el Ministerio de Salud de Ontario, desde hace varios años. El propósito de estas guías, desde su inicio, ha sido apoyar a las enfermeras proporcionándoles guías basadas en la evidencia para el cuidado del paciente en diversos contextos y sectores de la salud^19^. En el caso de FOSCAL, ha implementado guías específicas para valoración y manejo del dolor, prevención de caídas y lesiones derivadas de las caídas y riesgo de lesiones de piel por presión. Recientemente en un artículo publicado sobre la sostenibilidad de este modelo, sus autoras refieren una adherencia a la aplicación de las guías del 89%. No existen datos publicados aún de la eficacia de este modelo en relación con indicadores de calidad^20^.

Definitivamente, ofrecer una gestión adecuada del cuidado de enfermería en todas las fases del ciclo vital es un desafío significativo, donde la investigación en enfermería en las diferentes áreas de desempeño profesional es fundamental para fortalecer la práctica basada en la evidencia y la eficacia en los procesos de cuidado, en este sentido otra institución que desarrollado aportes a la gestión es la Fundación Cardiovascular de Colombia (FCV) ha publicado validación de instrumentos para aportar con la cultura de la calidad y seguridad del paciente^21^^,^^22^.

## Necesidades de Gestión del Cuidado Identificadas en la Región

Una de las necesidades más sentidas en la gestión del cuidado tiene que ver con la educación para la salud dirigidas al paciente y su familia, continuar con la innovación en este tipo de intervenciones sigue estando a la vanguardia, ya que a pesar de los esfuerzos que se realizan constantemente en este tipo de temas, el desconocimiento ha sido identificado hasta en un 80% de los usuarios de una misma institución^23^. De igual manera, esta situación ha sido estudiada en los mismos profesionales de enfermería, donde por ejemplo en temas de derecho se han encontrado niveles de conocimientos deficiente sumado a la carencia de procesos de formación^24^. En la profesión de enfermería, el cuidado se basa en un acto comunicativo que implica la aplicación de conocimientos científicos y tecnológicos, así como la comprensión del contexto sociocultural en el que se brinda atención a los pacientes y es aquí donde emerge procesos sustantivos de actualización constante siendo prioritario avanzar en formación pos gradual de alto nivel: maestrías y doctorados.

De otra parte, durante el ejercicio de la enfermería se han de develar los procesos de organización, supervisión, evaluación y promoción de la calidad del cuidado, donde la percepción del desempeño de enfermería, ha sido un tema que ha tomado relevancia en los últimos años. En relación a esto, algunos estudios han buscado identificar fortalezas y debilidades como en el estudio de Valdivieso que, si bien es antiguo, es una de las primeras caracterizaciones que sobre los profesionales de enfermería hay en Santander. En él, se señalaba en su mayoría una actitud favorable de las enfermeras del país, en especial aquellas de la región oriente, sur oriente y costa, hacia su ejercicio profesional. Sin embargo, la percepción desfavorable, tiene que ver como falta de reconocimiento social al igual que una actitud desfavorable hacia la autonomía y hacia la organización gremial en el país^25^. Esto deja en evidencia la necesidad apremiante de realizar acciones que favorezcan el liderazgo y cambio a nivel, gremial e institucional, implementando estrategias de cuidado con bases disciplinares favoreciendo los procesos en la mejora de la calidad en salud.

Lo anterior, se viene mencionando desde hacer varios años. En 2007 Beatriz Carvallo, en su momento presidenta de la junta directiva de la Asociación Nacional de Enfermeras de Colombia ANEC, mencionó durante el Congreso Nacional de enfermería sobre el impacto de las reformas de la seguridad social sobre las condiciones socio laborales para enfermería. Desde ese entonces se planteaban situaciones que se han convertido en retos como lo es la disminución de la autonomía de la enfermería y la desaparición de problemas de los departamentos de enfermería, la sustitución de funciones asistenciales por administrativas y la falta de reconocimiento de la recomendación de la OMS para fortalecer la enfermería. Esto constituye el verdadero reto para la gestión del cuidado en enfermería. Respecto a este escenario, se han descrito trabajos regionales en relación con la carga laboral de los profesionales de enfermería, en donde se hace evidente la necesidad de evaluar y analizar la distribución del recurso humano en estas unidades^26^. Estos temas toman relevancia pues Santander es un referente en salud en el Nororiente Colombiano y Suramericano pues el turismo en salud hace parte importante de la economía de la región por lo que enfermería debe aportar al desarrollo social desde el que hacer disciplinar.

Desde el punto de vista cualitativo, en algunos escenarios como los servicios de hospitalización en donde se hicieron evidentes los comentarios negativos sobre la gestión del cuidado humanizado. Se puede mejorar la atención de enfermería al dedicar más tiempo a escuchar a los pacientes, proporcionar información sobre los cuidados y estado de los pacientes, brindar atención oportuna, mejorar el estado de ánimo del profesional de enfermería y atender adecuadamente durante el horario nocturno^27^. Estos resultados argumentan la necesidad de publicar todo lo que desde la gestión se planea y se evalúa, para así validar el liderazgo que desde los departamentos y gerencias de enfermería se vienen haciendo. Sin embargo, es evidente que muchas de estas dificultades, tienen que ver con la falta de implementación de modelos de cuidado propios de enfermería.

Respecto a este liderazgo, en un estudio realizado en 2010 se describió en un grupo de 107 enfermeras de dos instituciones de salud en Bucaramanga, a través del instrumento de Hersey y Blanchard, un estilo de liderazgo predominante en guiar y participar que significa que existe un alto interés en las personas y bajo interés en las tareas de la producción respectivamente. La autora destaca la importancia del liderazgo en la formación de enfermería, con el fin de integrar habilidades como la toma de decisiones, liderazgo, comunicación, educación continua y administración. De esta manera, con las habilidades y formación seguirán siendo de gran valor en las instituciones de salud^28^.

Finalmente, el objetivo de enfermería es cuidar; el objetivo de la gerencia es la conducción de la producción de bienes o servicios en las organizaciones^8^. De esta manera, se puede inferir que la gestión de cuidado, cobra importancia siendo un desafío el liderazgo y la habilidad en la toma de decisiones basadas en la evidencia.

# Translation: Gestáo de Enfermagem no Santander: autoconhecimento ou administrado de saúde?

As necessidades de saúde continuam sendo uma questáo de interesse no desenvolvimento integral dos países. É prioritário estabelecer acóes para responder aos novos cenários e desafios ambientais, políticos e sociais do mundo atual. A gestáo do cuidado de enfermagem estabelece um processo de lideranca dentro das intervencóes com base nos cenários propostos pela Organizado Mundial da Saúde (OMS) e pela Organizado Pan-Americana da Saúde (OPAS)^1^ para esse desenvolvimento.

Existem várias definicóes de gestáo do cuidado, sendo que uma delas a define como a capacidade de planejar, organizar, motivar e controlar a prestacáo de cuidados oportunos, seguros e abrangentes que garantam a continuidade do cuidado e se baseiem em diretrizes claras para atingir o objetivo final de melhorar a saúde das pessoas^2^; também visa adaptar o cuidado as necessidades da pessoa, as demandas dos familiares, dos cuidadores e da comunidade^3^^,^^4^. Isso inclui o trabalho em conjunto, em que a consulta entre colegas profissionais de enfermagem gera redes com participacáo ativa no avanco do conhecimento e na colaboracáo interdisciplinar, obtendo um impacto na qualidade do servico que estáo prestando, além de reforcar o potencial e enriquecer o aprendizado significativo na prática profissional^5^.

Ao longo do tempo, a disciplina de enfermagem evoluiu em seu conhecimento, passando a adotar uma abordagem sistemática passo a passo, gerando sua própria estrutura de acáo denominada "Processo de Assistencia de Enfermagem" (PAE), uma ferramenta que comprovadamente contribuí de forma eficaz para a administrado de recursos para promover a gestáo do cuidado. Além disso, faz uso da enfermagem baseada em evidencias (EBE) como recurso para tomar as melhores decisóes em acóes de promodo da saúde, diagnóstico, tratamento, reabilitacáo ou cuidados paliativos.

## Administrado de saúde e gerenciamento de cuidados

A luz da gestáo organizacional, o objetivo fundamental é estabelecer modelos de gestáo nas empresas que possibilitem oferecer produtos ou servicos que satisfacam as necessidades dos usuários em um ambiente competitivo. Nesse sentido, náo basta apenas executar as tarefas corretamente; é necessário superar a concorrencia por meio da implementacáo de novas estratégias inovadoras a partir da gestáo da organizacáo^6^.

A gestáo do atendimento representa um grande desafio em termos de conhecimento gerencial e administrativo que é regido por regulamentos. Para obter um atendimento de qualidade, é necessário ter competencias científicas em termos de atendimento e processos a serem gerenciados. O gerenciamento do atendimento implica competencia em administracáo e requer julgamento crítico, visáo, previsáo e capacidade de resposta a mudancas rápidas na saúde e na doenca, bem como o uso eficaz de informacóes para cooperacáo e coordenacáo do trabalho. Na enfermagem, o desenvolvimento dessa capacidade gerencial adequada baseia- se em habilidades para gerenciar recursos, planejar cuidados, coordenar, supervisionar e monitorar^7^^,^^8^^,^^9^.

Devemos considerar estratégias para melhorar a assistencia de enfermagem e garantir a continuidade da assistencia centrada no paciente por meio do desenvolvimento de modelos organizacionais e práticas clínicas. Para isso, é importante implementar diferentes ferramentas para garantir a qualidade, e uma metodologia útil é o processo de enfermagem como um método científico para a elaboracáo e o desenvolvimento de planos de cuidados.

Há mais de cem anos, a enfermagem comecou a desenvolver sua base teórica por meio de estudos de pesquisa. Florence Nightingale refletia sobre suas observacóes, coletava e analisava dados nos hospitais em que trabalhava para melhorar os servicos de saúde. No entanto, foi somente na segunda metade do século passado que a enfermagem demonstrou seu estudo sistemático por meio de pesquisas rigorosas e da formulacáo de vários modelos teóricos e teorias que sustentam a prática^10^.

Portanto, a compreensáo dos modelos conceituais de enfermagem pode ser útil na prática de enfermagem, pois nos permite organizar nossos pensamentos sobre os diferentes aspectos de uma situacáo prática. Além disso, o uso de terminologia comum entre os profissionais pode facilitar uma comunicacáo significativa. Ele também pode servir de guia na prática de cuidados, ensino e pesquisa^11^.

Assim, para que o gerenciamento do cuidado de enfermagem seja mais abrangente e holístico, é necessário entender e apreciar: as estruturas usadas no gerenciamento do cuidado e como elas orientam o desenvolvimento profissional e disciplinar. Prestar atencáo aos desafios presentes no ambiente ajuda as profissóes a manter um atendimento atualizado e de qualidade, mas, acima de tudo, com base em fundamentos sólidos.

Nesse sentido, a partir da teoria do autocuidado de Orem, sáo destacados os elementos relevantes para o cuidado de enfermagem. A proposta busca direcionar e gerenciar o cuidado de enfermagem, com o objetivo de auxiliar o individuo a alcanzar e manter suas acóes de autocuidado, favorecendo, assim, a saúde e a vida, a recuperado da doenca e a adaptado aos seus efeitos^12^^,^^13^^,^^14^. Dentro dessa estrutura disciplinar, quatro teóricas de enfermagem: Imogene King, Virginia Henderson, Dorothea Orem e Jean Watson, incluem em seus postulados elementos que orientam a fundo gerencial. Na abordagem de Imogene King, a instituido hospitalar representa o fator ambiental mais relevante para a pessoa que recebe cuidados institucionais. Para Henderson, o reconhecimento das necessidades individuais é fundamental. No caso de Orem, além do que foi mencionado acima, é essencial garantir a disponibilidade de recursos adequados para o atendimento. Watson argumenta que a abordagem de enfermagem está intrinsecamente ligada ao cuidado humanizado em todas as áreas da prática profissional, incluindo administrado, gerenciamento, ensino, servido e pesquisa^15^.

O reconhecimento das habilidades do profissional de enfermagem contribui para a construcáo de um paradigma científico diferente, desafiando o medo e assumindo riscos diante dos desafios do cuidado.

## Ferramentas em Gestáo de Cuidados em Santander

A enfermagem baseada em evidencias contribui para a demanda por novas abordagens de gerenciamento orientadas para o cuidado personalizado com base na experiencia individual de saúde. A seguir, apresentamos uma descrido dos avanzos encontrados na literatura sobre gerenciamento de cuidados no departamento de Santander.

Uma das primeiras publicares sobre a aplicado de ferramentas de enfermagem, como o processo de enfermagem, foi feita em 1997, quando um estudo descritivo estabeleceu dados sobre o conhecimento e a aplicabilidade dos profissionais de enfermagem em Bucaramanga e sua área metropolitana, e constatou que apenas 25% dos enfermeiros se lembravam corretamente das etapas. Foram descritas limitacóes importantes para a implementacáo do processo de enfermagem, como o tempo necessário para sua aplicado e registro, a falta de consenso na elaboracáo do diagnóstico, a falta de compromisso institucional para adotar o processo como ferramenta de trabalho, a falta geral de conhecimento do processo pela equipe, a ausencia de sistemas de registro específicos e a sobrecarga de pacientes em relacáo ao número de enfermeiros disponíveis. Os autores do artigo propuseram solucóes para superar essas limitacóes, incluindo programas de educacáo continuada, a unificacáo de critérios entre profissionais e professores, o aumento do número de enfermeiros nas instituicóes, o apoio institucional para a criacáo de sistemas de registro específicos e maiores recursos financeiros para as instituicóes de saúde^16^.

Posteriormente, 24 anos depois, Lesmes, em 2018, estudou os fatores relacionados a aplicado do processo de enfermagem, no qual 59% dos participantes expressaram seu uso. No entanto, 98% relataram a falta de tempo como um fator para náo o aplicar. Foi encontrada uma relacáo estatisticamente significativa entre a aplicacáo do processo de cuidado de enfermagem e sua percepcáo como uma ferramenta facilitadora do cuidado, bem como a capacidade do profissional de enfermagem de relacioná-lo a teoria aprendida na universidade. Diferentemente de estudos anteriores, essas associacóes náo se limitaram as condicóes de trabalho ou académicas. Os resultados sugerem a importancia da aplicado da teoria na prática e da avaliacáo de sua implementacáo em uma variedade de ambientes profissionais^17^.

Em 2008, o Hospital Universitário de Santander publicou um dos primeiros relatórios descrevendo processos de melhoria na gestáo de cuidados. O estudo apresentou os resultados da implementacáo de um modelo de garantia de qualidade na assistencia de enfermagem, cujo objetivo era detectar pontos fortes e fracos no processo de assistencia de enfermagem, propor medidas corretivas, preventivas e de melhoria e desenvolver novas abordagens de assistencia baseadas em evidencias para fazer as mudanzas adequadas. Esse processo consistía na elaborado de processos e protocolos de enfermagem que incluíam tópicos como registros de enfermagem, lavagem das máos, cateterizado venosa periférica, eventos adversos e entrada e saída de pacientes. Com essa implementaáo, eles concluíram que conseguiram melhorar a qualidade do servido, a satisfago do paciente e da familia, o uso eficiente dos recursos, a otimizaáo do trabalho dos enfermeiros e assistentes hospitalares para fortalecer os processos de ensino e assistencia^18^.

A Fundación Oftalmológica de Santander Clínica Carlos Ardila Lulle (FOSCAL) foi considerada outra instituido que implementou processos de melhoria da gestáo de cuidados ao adotar algumas das Diretrizes de Boas Práticas (Programa BPG) da RNAO (Registered Nurses' Association of Ontario) financiadas pelo Ministério da Saúde de Ontário por vários anos. O objetivo dessas diretrizes, desde sua criado, tem sido apoiar os enfermeiros fornecendo diretrizes baseadas em evidencias para o atendimento ao paciente em uma variedade de ambientes e setores de saúde^19^. No caso da FOSCAL, ele implementou diretrizes específicas para avaliaáo e gerenciamento da dor, prevendo de quedas e lesoes resultantes de quedas e risco de lesoes cutáneas por pressáo. Em um artigo publicado recentemente sobre a sustentabilidade desse modelo, os autores relatam uma adesáo de 89% a aplicado das diretrizes. Ainda náo há dados publicados sobre a eficácia desse modelo em relado aos indicadores de qualidade^20^.

Nesse sentido, outra instituido que tem desenvolvido contribuyes para a gestáo é a Fundado Cardiovascular da Colombia (FCV), que publicou a validado de instrumentos para contribuir com a cultura da qualidade e da seguranza do paciente^21^^,^^22^.

## Necessidades de gerenciamento de cuidados identificadas na regiáo

Uma das necessidades mais sentidas na gestáo do cuidado tem a ver com a educado em saúde para os pacientes e suas famílias. Continuar inovando nesse tipo de intervendo ainda é uma prioridade, já que, apesar dos esforos que sáo feitos constantemente nesse tipo de assunto, a falta de conhecimento foi identificada em até 80% dos usuários da mesma instituido^23^. Da mesma forma, essa situado foi estudada entre os próprios profissionais de enfermagem, onde, por exemplo, foram encontrados níveis deficientes de conhecimento no campo do direito, além da falta de processos de treinamento^24^. Na profissáo de enfermagem, o cuidado se baseia em um ato comunicativo que envolve a aplicado de conhecimentos científicos e tecnológicos, bem como a compreensáo do contexto sociocultural em que o cuidado ao paciente é prestado, e é aí que emergem processos substantivos de atualizaáo constante, com prioridade para o avanzo na formado de pós-graduaáo de alto nível: mestrado e doutorado.

Por outro lado, durante a prática da enfermagem, devem ser desvendados os processos de organizado, supervisáo, avaliaáo e promodo da qualidade da assistencia, onde a percepdo do desempenho da enfermagem tem sido um tema que se tornou relevante nos últimos anos. Em relado a isso, alguns estudos tem buscado identificar pontos fortes e fracos, como o estudo de Valdivieso, que, apesar de antigo, é uma das primeiras caracterizaóes dos profissionais de enfermagem em Santander. Nesse estudo, a maioria dos enfermeiros do país, especialmente os das regioes leste, sudeste e litoránea, tinha uma atitude favorável em relado a sua prática profissional. Entretanto, a percepdo desfavorável está relacionada a falta de reconhecimento social, bem como a uma atitude desfavorável em relado a autonomia e a organizado profissional no país^25^. Isso destaca a necessidade urgente de aóes que favoream a mudanza de liderana em nível sindical e institucional, implementando estratégias de atendimento com base disciplinar, favorecendo processos para melhorar a qualidade da saúde.

Esse fato vem sendo mencionado há vários anos. Em 2007, Beatriz Carvallo, na época presidente da diretoria da Associacáo Nacional de Enfermeiros da Colombia (ANEC), mencionou durante o Congresso Nacional de Enfermagem o impacto das reformas da previdencia social sobre as condicóes sociais e de trabalho dos enfermeiros. Desde entáo, surgiram situacóes que se tornaram desafios, como a diminuido da autonomia da enfermagem e o desaparecimento dos problemas nos departamentos de enfermagem, a substituido das funcóes de cuidado por funcóes administrativas e a falta de reconhecimento da recomendacáo da OMS para fortalecer a enfermagem. Isso constitui o verdadeiro desafio para o gerenciamento da assistencia de enfermagem. Em relacáo a esse cenário, foram descritos estudos regionais sobre a carga de trabalho dos profissionais de enfermagem, nos quais fica evidente a necessidade de avaliar e analisar a distribuicáo dos recursos humanos nessas unidades^26^. Essas questóes ganham relevancia pelo fato de Santander ser um ponto de referencia para a saúde no Nordeste da Colombia e na América do Sul, pois o turismo de saúde é parte importante da economia da regiáo, e a enfermagem deve, portanto, contribuir para o desenvolvimento social do ponto de vista disciplinar.

Do ponto de vista qualitativo, em alguns cenários, como os servicos de hospitalizacáo, foram evidenciados comentários negativos sobre a gestáo do atendimento humanizado. O atendimento de enfermagem pode ser melhorado dedicando-se mais tempo para ouvir os pacientes, fornecendo informacóes sobre o atendimento e a condicáo dos pacientes, fornecendo atendimento oportuno, melhorando o humor do profissional de enfermagem e fornecendo atendimento adequado durante as horas noturnas^27^. Esses resultados defendem a necessidade de publicar tudo o que é planejado e avaliado do ponto de vista da gerencia, a fim de validar a lideranca que os departamentos de enfermagem e a gerencia estáo oferecendo. No entanto, é evidente que muitas dessas dificuldades tem a ver com a falta de implementacáo de modelos de assistencia de enfermagem.

Com relacáo a essa lideranca, um estudo realizado em 2010 descreveu em um grupo de 107 enfermeiros de duas instituicóes de saúde de Bucaramanga, por meio do instrumento de Hersey e Blanchard, um estilo de lideranca predominante em orientar e participar, o que significa que há um alto interesse nas pessoas e baixo interesse nas tarefas de producáo, respectivamente. O autor destaca a importancia da lideranca na educacáo em enfermagem, a fim de integrar habilidades como tomada de decisóes, lideranca, comunicacáo, educacáo continuada e gerenciamento. Dessa forma, as habilidades e o treinamento continuaráo a ser de grande valor nas instituicóes de saúde^28^.

Por fim, o objetivo da enfermagem é cuidar; o objetivo da administracáo é conduzir a producáo de bens ou servicos nas organizacóes^8^. Dessa forma, pode-se inferir que a gestáo do cuidado se torna importante, sendo a lideranca e a capacidade de tomar decisóes baseadas em evidencias um desafio.
